# Supplementary material for: Transcript isoform sequencing reveals widespread promoter-proximal transcriptional termination in Arabidopsis
Source: Nat Commun. 2020 May 22;11:2589. doi: 10.1038/s41467-020-16390-7 (PMC7244574; doi:10.1038/s41467-020-16390-7)
Supplement: Supplementary file 1 — Supplementary Information [file 41467_2020_16390_MOESM1_ESM.pdf]

Supplementary Information

Transcript isoform sequencing reveals widespread promoter-proximal transcriptional termination in Arabidopsis

Thomas et al.

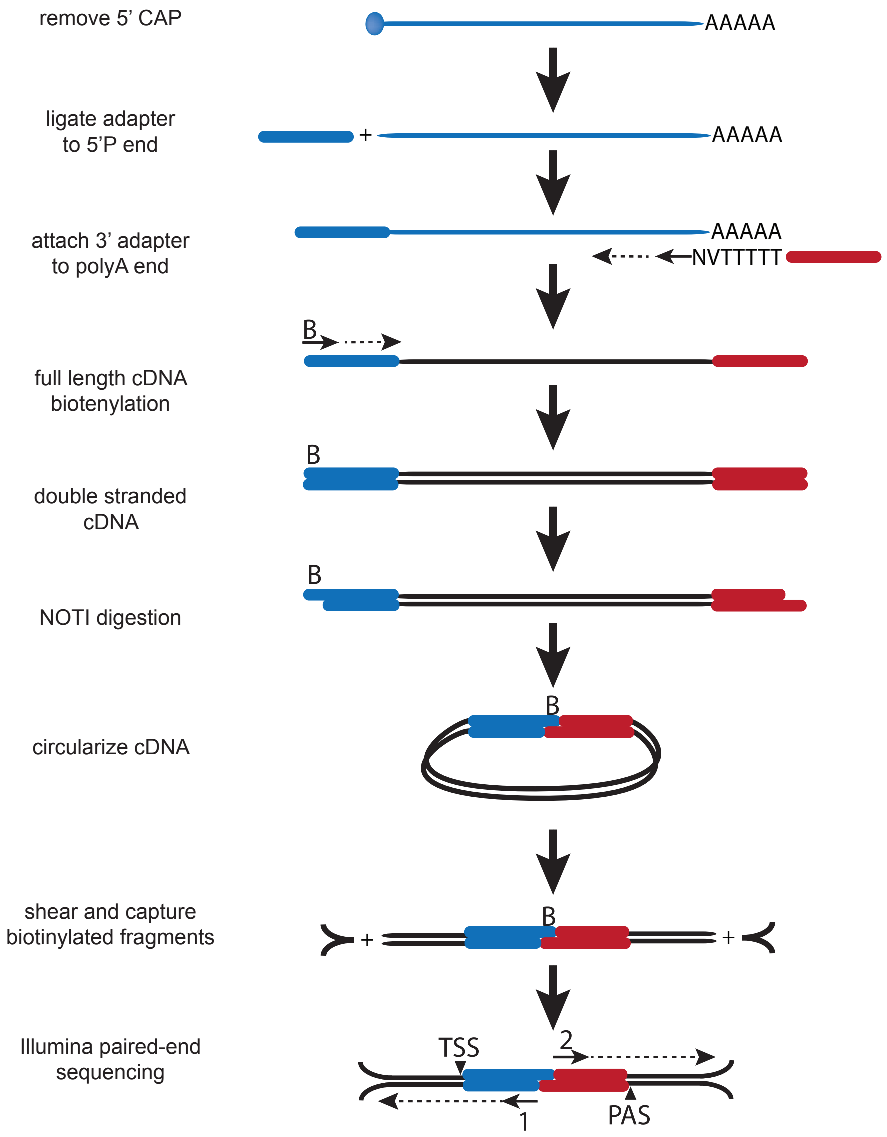

Supplementary Figure 1. Improved TIF-seq method workflow. Schematic representation of TIF-seq workflow (also see method section for details). A notable change to the original method include custom Illumina-compatible 5' and 3' adaptors for sequencing out of the insert. For other improvements to the protocol. The letter "B" indicates a biotin-group added during double-stranded cDNA synthesis step for streptavidin capture. First and second reads identify TIF-TSS and TIF-PAS, respectively.

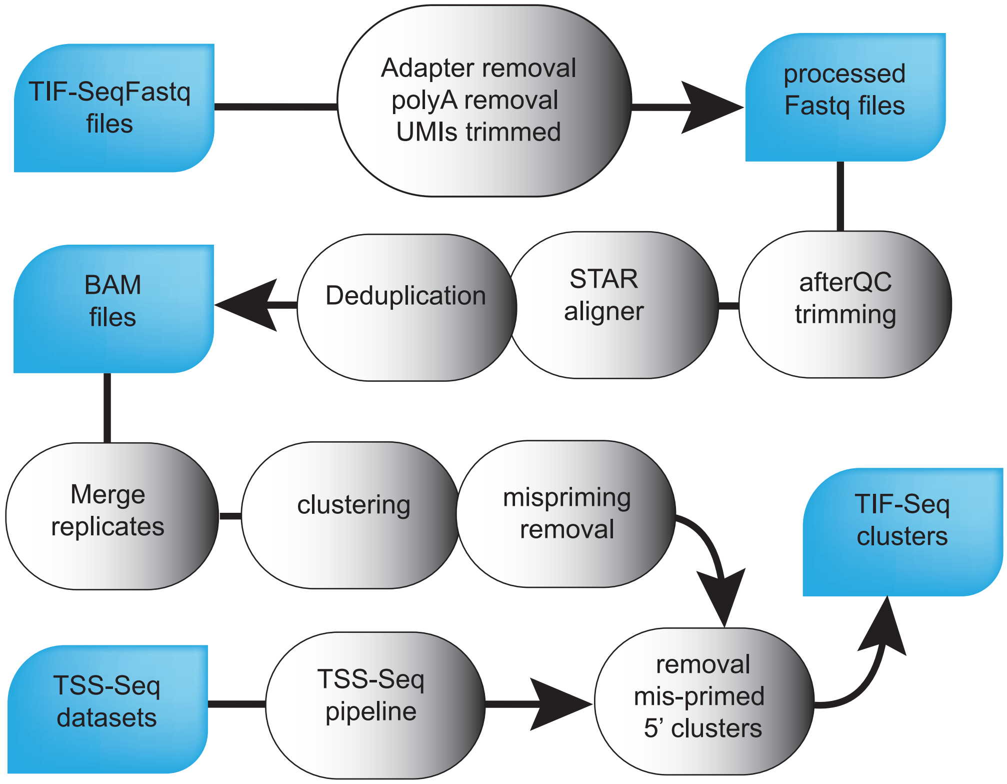

Supplementary Figure 2. Computational workflow for TIF-seq data. Schematic representation of workflow for handing TIF-seq data and calling TIF-clusters (see Methods). The pipeline for handling TSS-seq data has been previously described (1,2)

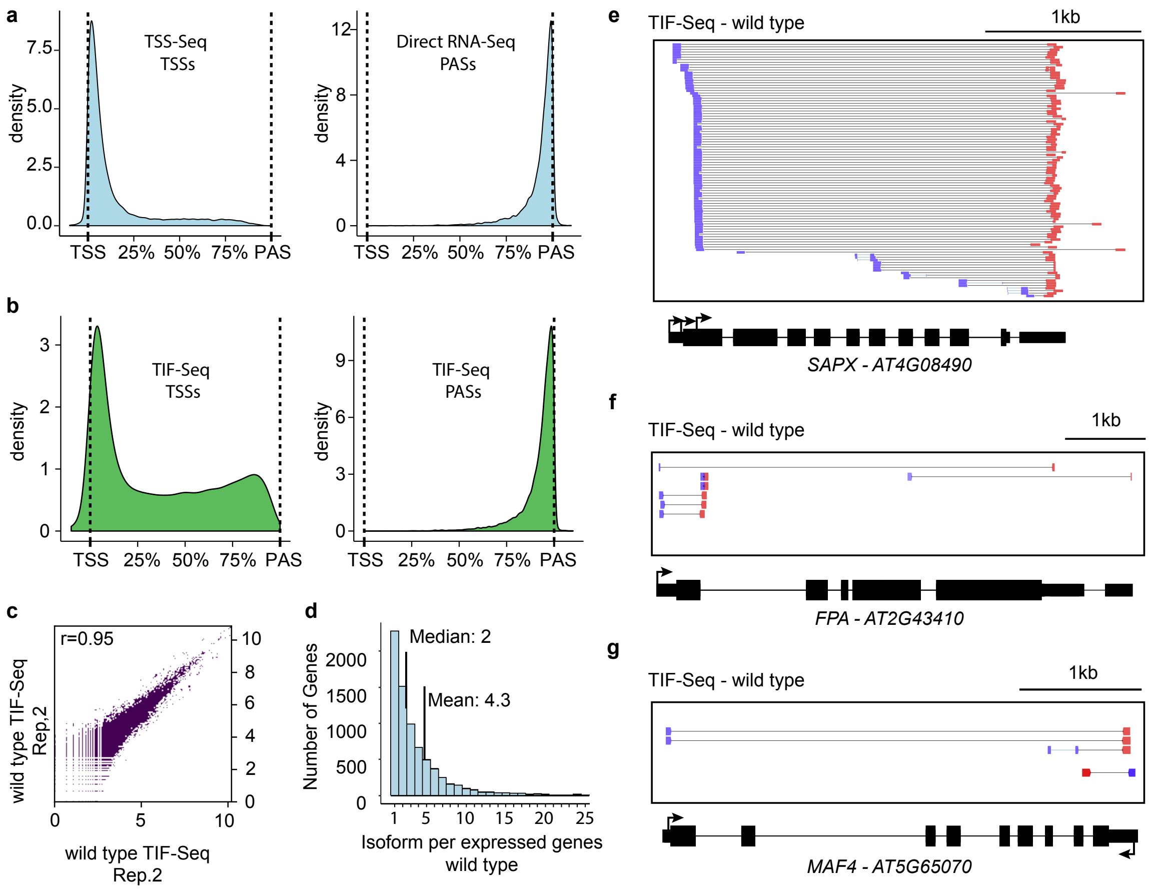

Supplementary Figure 3. TIF-seq control analyses for wild type validate characterized alternative gene isoforms. **a** Density plots for TSS positions from TSS-seq2 and TIF-seq (TIF-TSSs) and PAS positions from Direct RNA-seq and TIF-seq (TIF-PASs) (3). Bias of TIF-Seq to detect short molecules, and thus enriched on internal cryptic transcripts (TSS biased towards the 3´ end), has been previously reported (4). This can be corrected by excluding TIF-clusters without a TIF-TSS validated by a second method, such as TSS-seq. 75.8% of TIF-clusters in wild type were validated by TSS-seq, while the remaining 14.2% (mostly enriched towards genic 3'-ends) were discarded. See Supplementary Table 1 and Methods for more details. See Fig. 1c for the distribution of TIF-TSSs following this data processing step. **b** Density plot as in **(a)** with Direct-sequencing of PAS **c** Scatterplot of log10(coverage)/5 nucleotide bins for TIF-reads from two independent replicates in wild type (see Methods). R indicates the pearson correlation value between the two datasets. **d** Histogram of number of TIF-clusters reveals a mean of 4.3 TUs and a median of 2 TUs per expressed gene in wild type. **e-g** Genome browser screenshots of TIF-reads for previously characterized alternative gene isoforms stemming from **(e)** alternative TSSs at the *STROMAL ASCORBATE PEROXIDASE (SAPX)* gene (5), **(f)** alternative PAS at the *FLOWERING PROTEIN A (FPA)* gene (6), and **(g)** a recently characterized antisense transcript that regulates the *MADS AFFECTING FLOWERING 4 (MAF4)* gene (7). Gene model schematics are shown below the TIF-seq data screenshots. TSSs (blue) and PASs (red) identified by TIF-seq are illustrated.

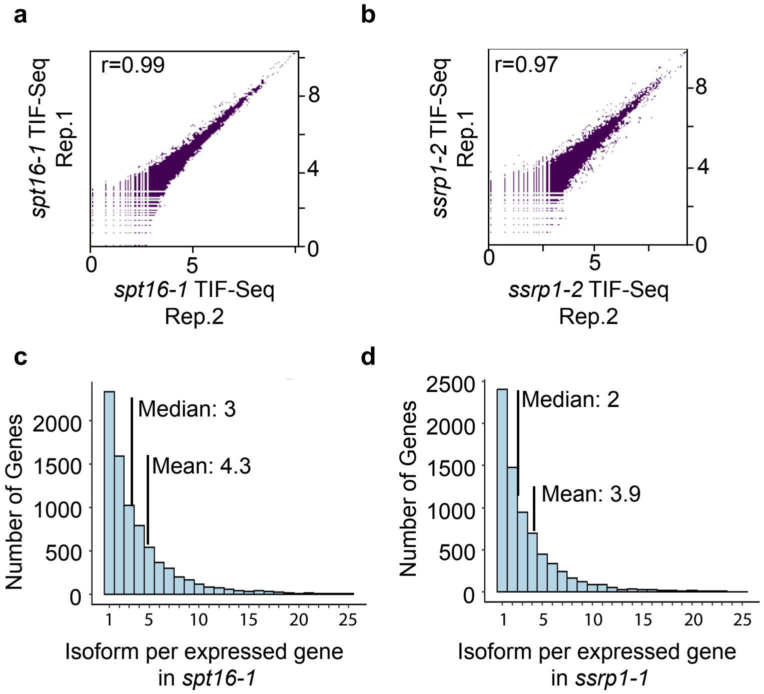

Supplementary Figure 4. TIF-seq analyses in fact mutants reveal co-transcriptional regulation of alternative gene isoforms. FACT consists of two subunits, Suppressor of Ty 16 (SPT16) and Structure-specific recognition protein 1 (SSRP1). **a** Scatterplot of log10(-coverage)/5 nt bins for TIF- reads from two independent replicates in FACT mutant *spt16-1* (see Methods). **b** Scatterplot of log10(coverage)/5 nt bins for TIF-reads from two independent replicates in FACT mutant *ssrp1-2.* **c** Histogram of number of TIF-clusters per expressed gene in *spt16-1* (mean: 4.3; median: 3). **d** Histogram of number of TIF-clusters per expressed gene in *ssrp1-2* (mean: 3.9; median: 2).

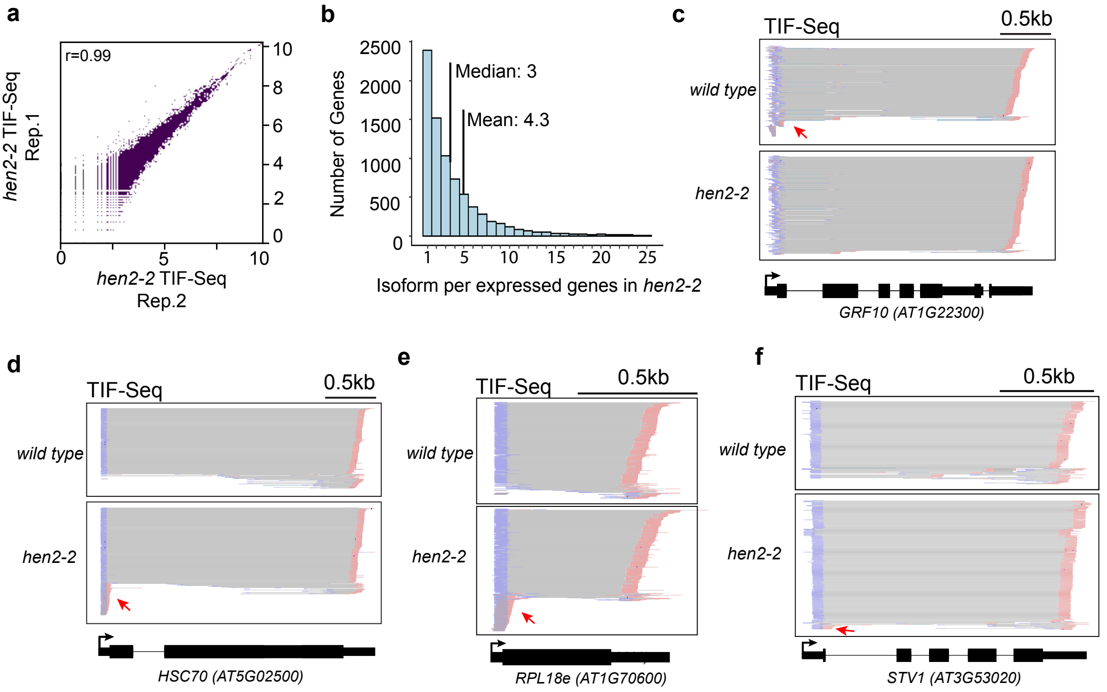

Supplementary Figure 5. TIF-seq analyses in *hen2-2* reveal cryptic isoform boundaries genome-wide in *Arabidopsis*. **a** Scatterplot of log10(coverage)/5 nt bins for TIF-reads from two independent replicates in *hen2-2* (see Methods). **b** Histogram of number of TIF-clusters per expressed gene in *hen2-2* (mean: 4.3; median: 3). **c-f** Genome browser screen-shots of TIF-reads for additional genes with detected sppRNA TIF-clusters used here to study premature-termination in *Arabidopsis*: **(c)** *GENERAL REGULATORY FACTOR 14 EPSILON* (*GRF10*; *AT1G22300*), **(d)** *HEAT SHOCK COGNATE PROTEIN 70-1* (*HSC70-1*; *AT5G02500*),**(e)** *RIBOSOMAL PROTEIN L18e/L15* (*RPL18e*; *AT1G70600*), **(f)** *RIBOSOMAL PROTEIN L24* (*STV1*; *AT3G53020*). Gene model schematics are shown below the TIF-seq data screenshots. TSSs (blue) and PASs (red) identified by TIF-seq are illustrated. Red arrows indicate termination near TSSs.

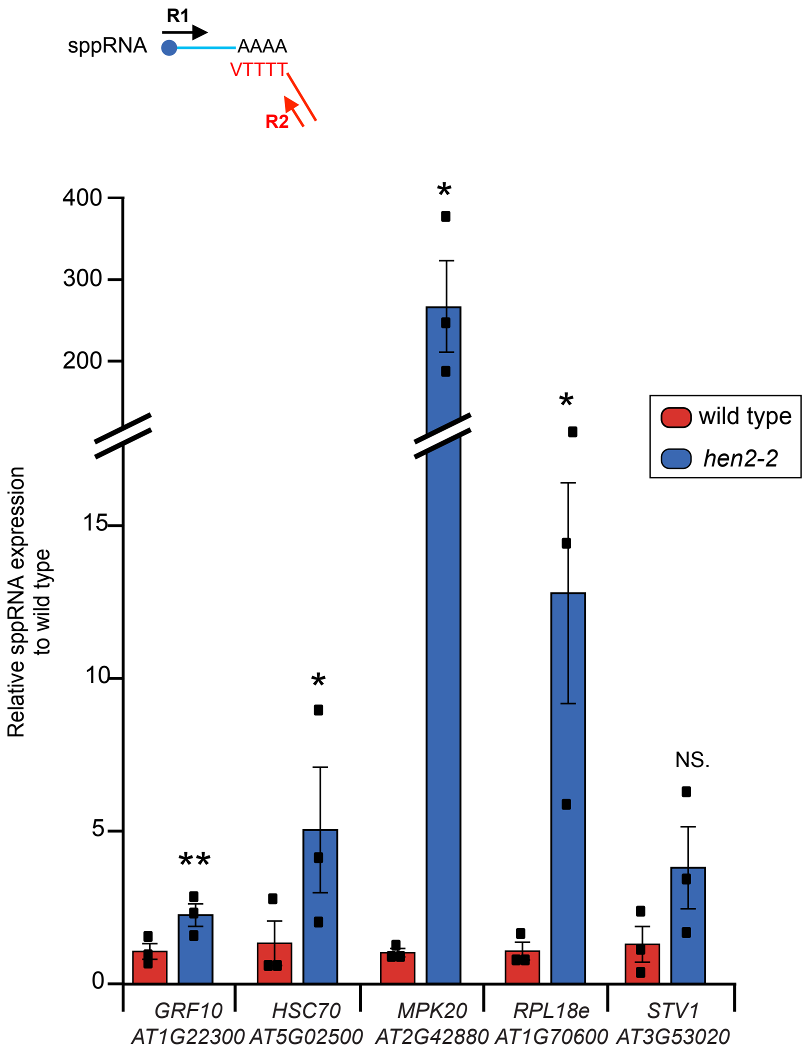

Supplementary Figure 6. Quantifying sppRNA levels in *hen2-2*. sppRNA expression in *hen2-2* relative to wild type by RT-qPCR for the 5 genes shown in Fig. 3g and Supplementary Fig. 5d-f. Data are presented as mean values +/- SEM from three independent experiments. Black dots indicate individual data points (n=3). Single asterisk denotes satistical significance between wild type and *hen2-2*, with p<0.05 by two-sided Student's t-test, two asterisks denote p<0.01 and NS denotes no statistically significant differences.The schematic on top of the bar plot represents the qPCR amplification procedure to detect sppRNAs where R1 and R2 for each genes are described in Supplementary Data 2. Source data are provided in the Source

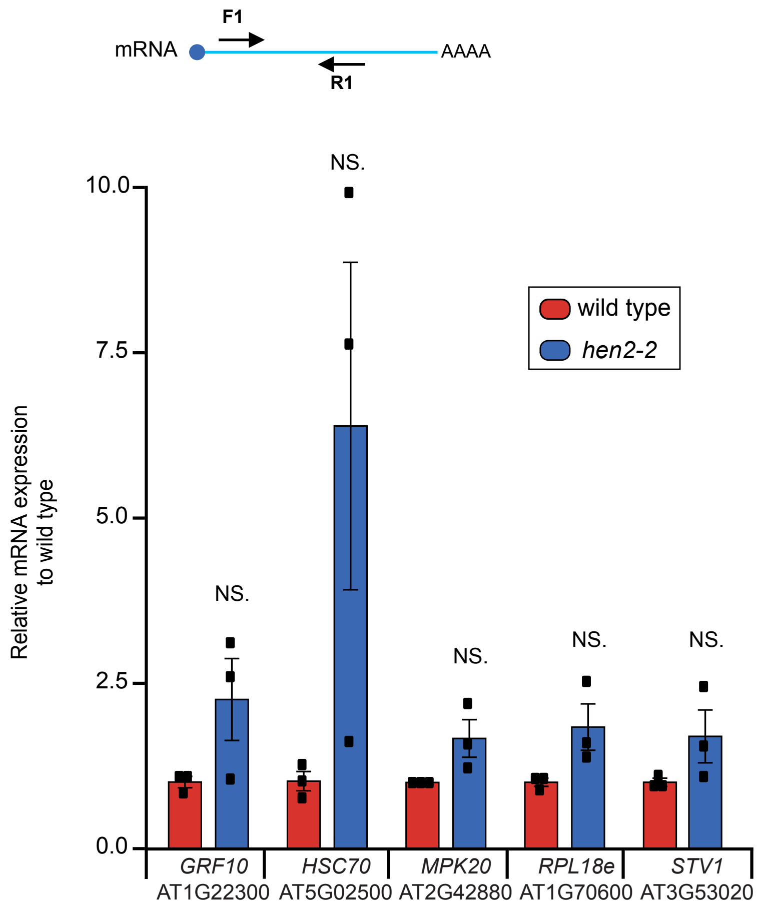

Supplementary Figure 7. Quantifying mRNA levels in *hen2-2*. mRNA expression in *hen2-2* relative to wild type by RT-qPCR for the 5 genes shown in Fig. 3g and Supplementary Fig. 5c-f. Data are presented as mean values +/- SEM from three independent experiments. Black dots indicate individual data points (n=3). NS denotes no statistically significant differences in relative expression compared to wild type by a two-sided Student's t-test. The schematic on top of the bar plot represents the qPCR amplification procedure to detect mRNA in relation with sppRNA specfic RT (see methods) where F1 and R2 for all genes are described in Supplementary Data 2. Source data are provided in the Source Data file.

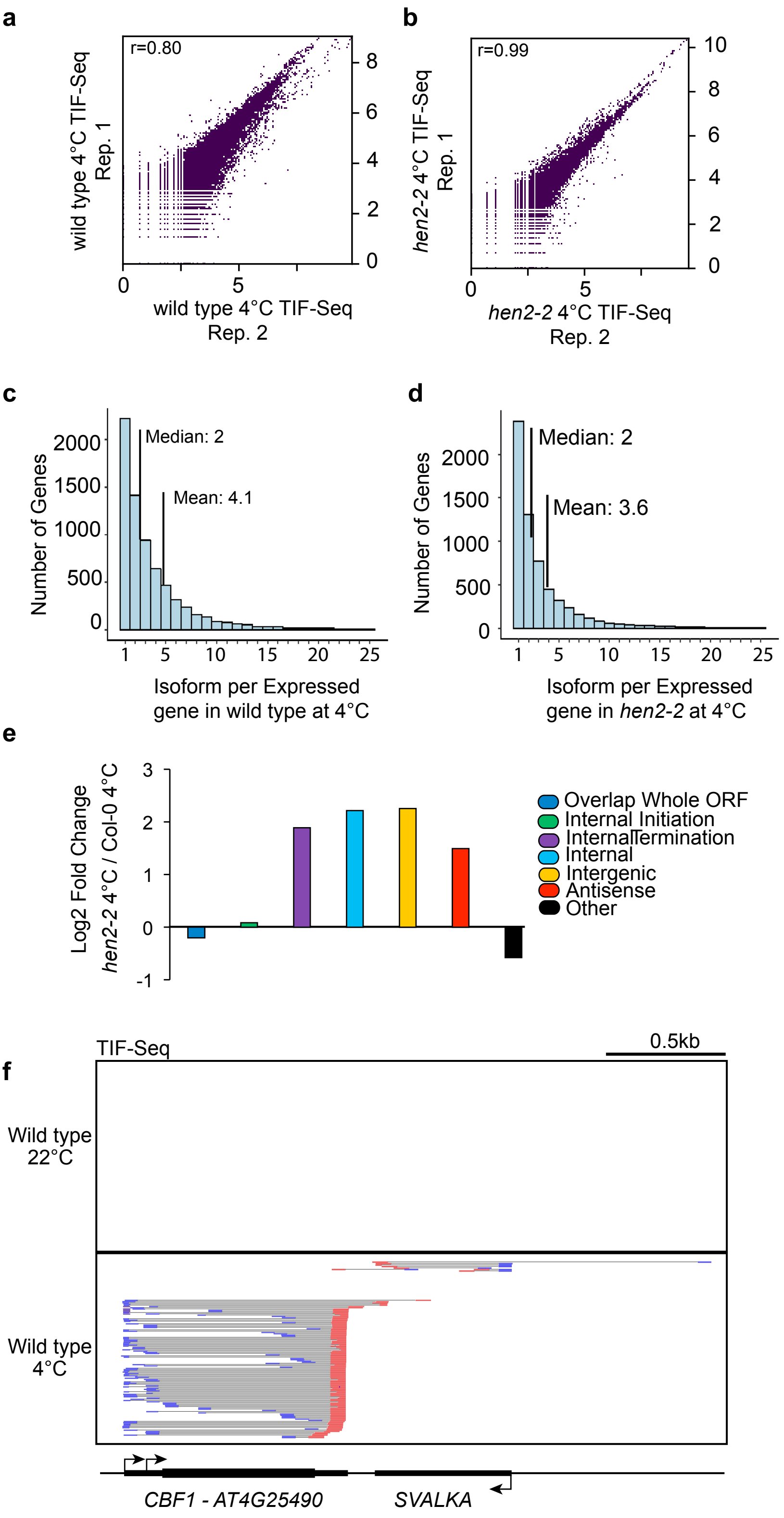

Supplementary Figure 8. TIF-seq analyses in cold-treated wild type and *hen2-2* mutants. **a** Scatterplot of log10(coverage)/5 nt bins for TIF-reads from two independent replicates in wild type cold-treated at 4°C. **b** Scatterplot of log10(coverage)/5 nt bins for TIF-reads from two independent replicates in *hen2-2* cold-treated at 4°C. **c** Histogram of number of TIF-clusters per expressed gene in cold-treated wild type (mean: 4.1; median: 2) **d** Histogram of number of TIF-clusters per expressed gene in cold-treated *hen2-2* (mean: 3.6; median: 2). **e** Log2 fold change of TIF cluster category proportions in cold-treated *hen2-2* compared to cold-treated wild type. **f** Genome browser screenshot depicting TIF-seq detection of cold-induced *C-REPEAT BINDING FACTOR 1* (*CBF1*) gene expression and both isoforms of the lncRNA *SVALKA*. (1) Source data of **(e)** are provided in the Source Data file.

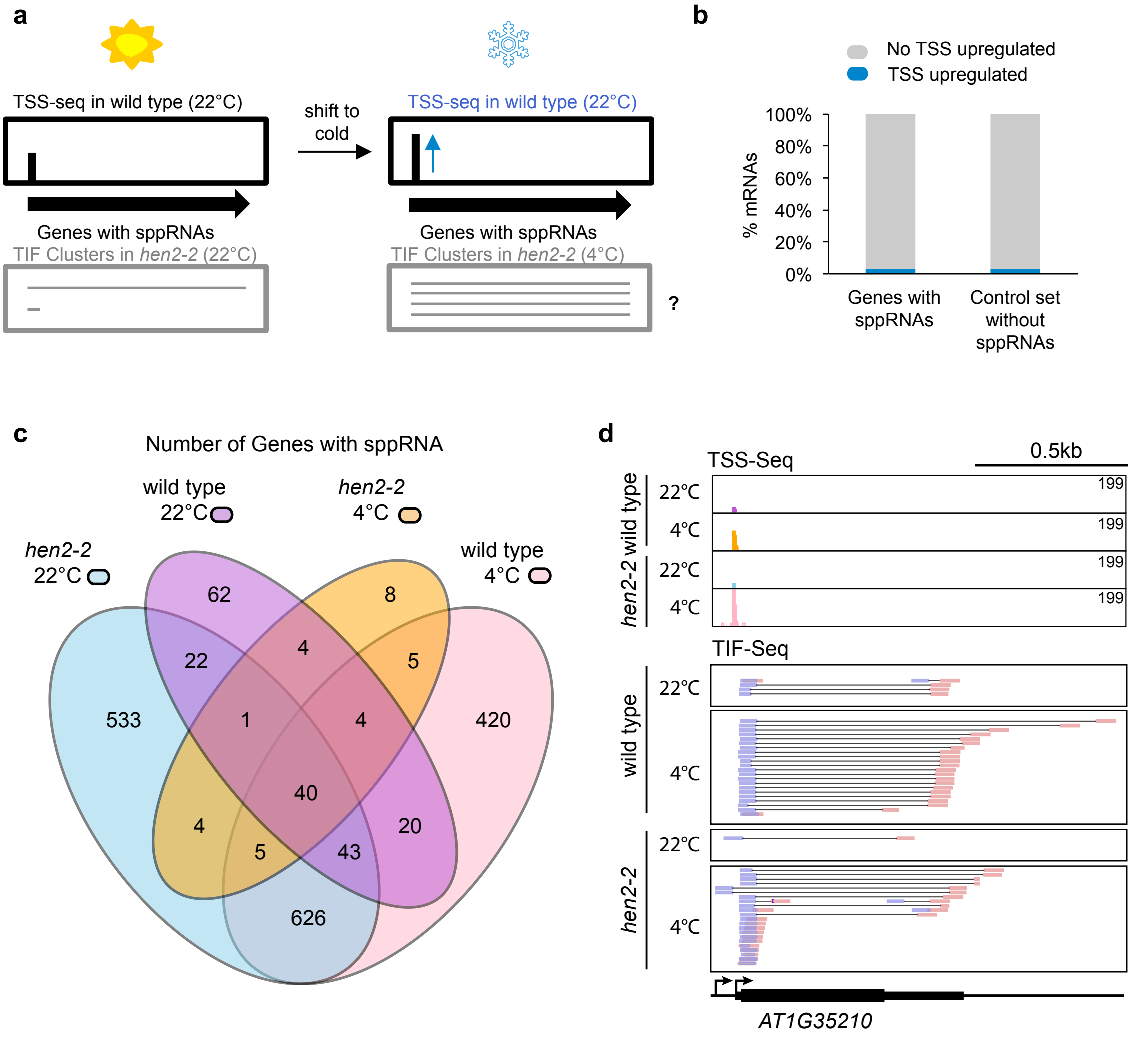

Supplementary Figure 9. Cold treatment stimulates sppRNA production at many induced genes. **a** Illustration of hypothetical gene expression regulation following environmental change by supporting elongation beyond the premature termination site of sppRNA. Genes may increase mRNA formation on the expense of sppRNA when the environment changes to regulate gene expression. Since such genes would represent cryptic sppRNA before the cold, we would expect genes matching that profile to show increased expression wild type TSS-seq data in the cold **b** Experimental test of hypothesis illustrated in **(a)**. Percentage of sppRNA genes that increase mRNA expression (TSS-seq data in wild type) following cold treatment compared to control set of genes without detectable sppRNAs. For both classes tested: n=1153. See methods. **c** Venn diagram depicting number of genes with detectable sppRNAs in wild type and/or hen2-2 before and after 3 hours of cold treatment. See Supplementary Data 2 and 3 for sppRNA gene lists and sppRNA genomic coordinates, respectively. **d** Genome browser screenshot of TSS- and TIF-seq at the *AT1G35210* gene in wild type and *hen2-2* both before and after cold treatment.Increased *AT1G35210* mRNA levels correspond with the elevated sppRNA levels following cold treatment. Source data of **(b-c)** are provided in the Source Data file.

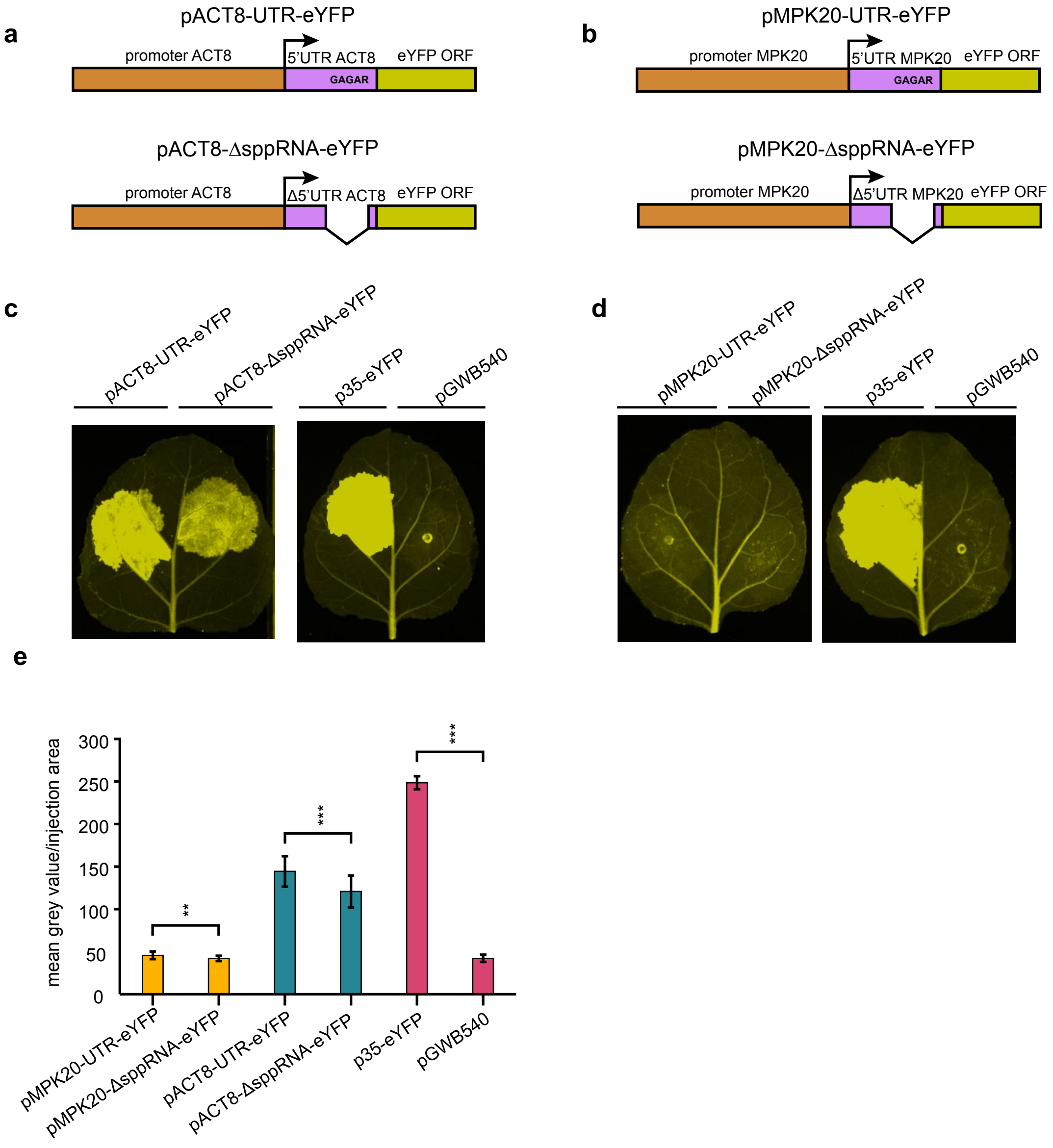

Supplementary Figure 10. Detection of the sppRNA termination reduces reporter gene activity. **a-b** Schematic representation of constructs used for *N. benthamiana* leaf injection with the pGWB540 plasmid. **(a)** The *ACT8* (*AT1G49240*) promoter includings 5'-UTR were fused to eYFP (pACT8-UTR-eYFP) and sppRNA site was deleted in a new construct (pACT8-Δ sppRNA-eYFP). **(b)** The *MPK20* (*AT2G42880*) promoter including 5'-UTR were fused to eYFP (pMPK20-UTR-eYFP) and sppRNA site was deleted in a new construct (pMPK20-ΔsppRNA-eYFP). **c** Pictures of injected *N. benthamiana* leafs with constructs in **(a)**, positive control (p35-eYFP) and negative control (pGWB540) empty vector. **d** Pictures of injected *N. benthamiana* leafs with *MPK20* constructs in **(b)**, positive control (p35-eYFP) and negative control (pGWB540) empty vector. **e** Measurements of grey values per injection area for contructs in **(a-b)**. Data are presented as mean +/- SEM from at least 15 independent experiments. Two asterisks denote $p<0.01$, while three asterisks denote $p<0.001$ between mutant and wild type by two sided Student's t-test. Source data of **(e)** are provided in the Source Data file.

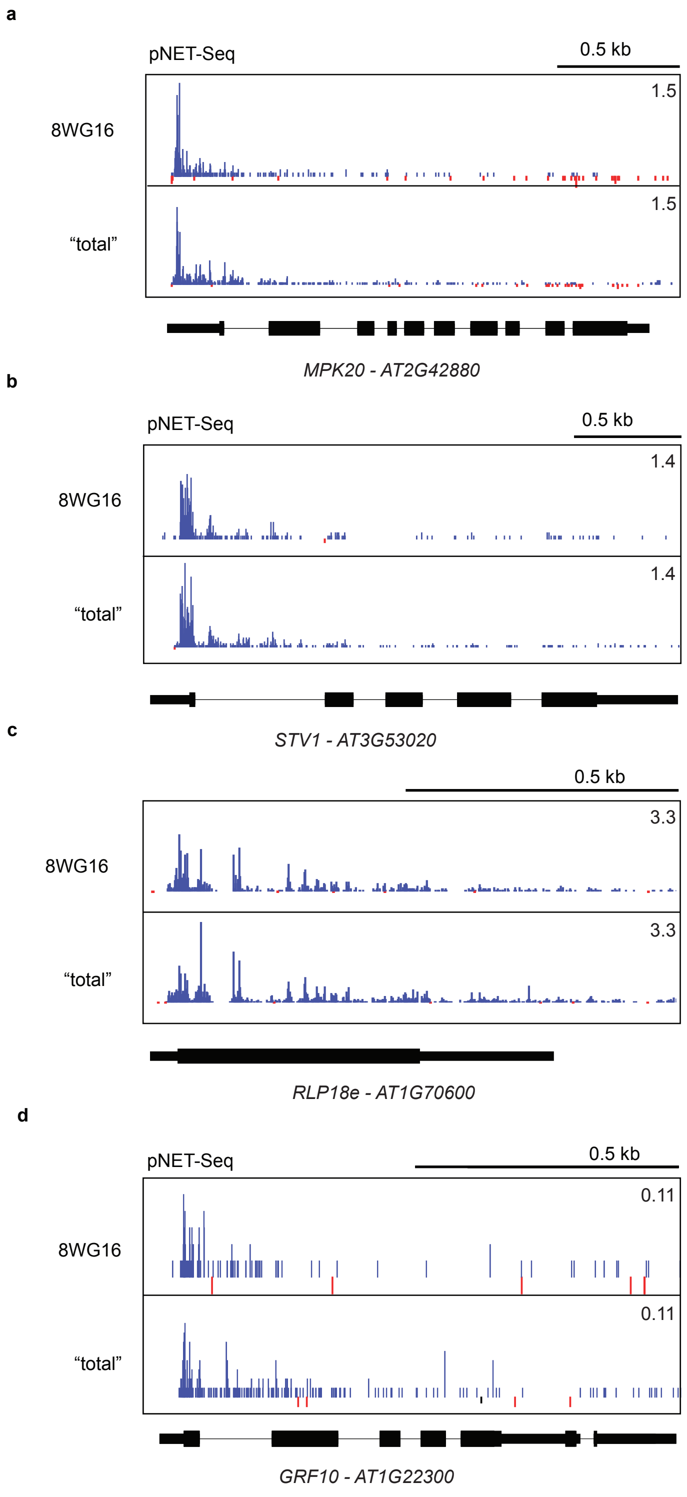

Supplementary Figure 11. Promoter-proximal RNAPII peaks at genes with sppRNAs. Genome browser screenshots of pNET-seq data from experiments using different RNAPII antibodies, 8WG16 and “Total” RNAPII (8), along sppRNA-containing genes: **a** *MPK20* (*AT2G42880*), **b** *RPL24* (*AT3G53020*), **c** *RPL18e* (*AT1G70600*), and **d** *GRF10* (*AT1G22300*). Blue reads on the positive (sense) strand. Red reads on the negative (antisense) strand.

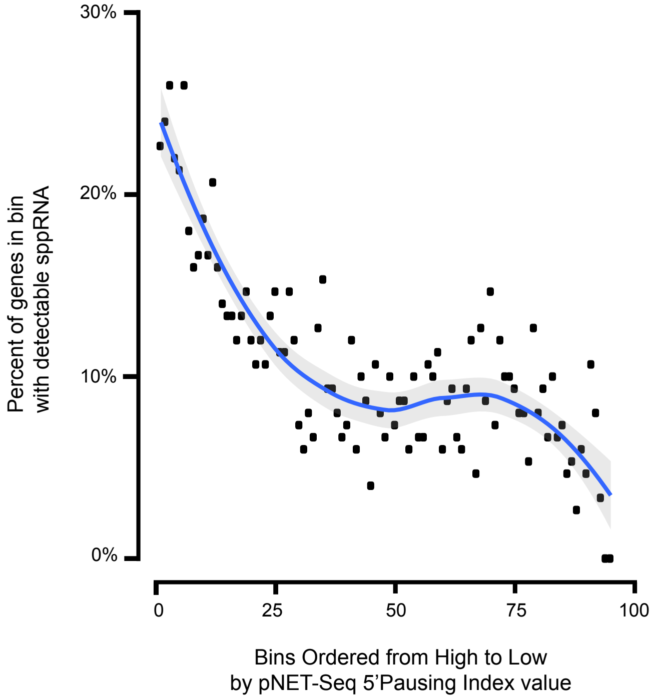

Supplementary Figure 12. Increased promoter-proximal RNAPII stalling strongly correlates with sppRNA production. "5' pausing index" values were calculated for 14,126 of the 75% most highly expressed genes from pNET-seq (8WG16) (8) (see Methods). The percentage of genes that have detectable sppRNAs per bin of genes (150 genes per bin) are represented as individual black dots. The smoothed trend line is indicated in blue, while shading represents a confidence 95% interval on the smoothing curve calculated with iteratively reweighted least squares. Overall, higher levels of promoter-proximal RNAPII stalling strongly correlates with sppRNA formation.

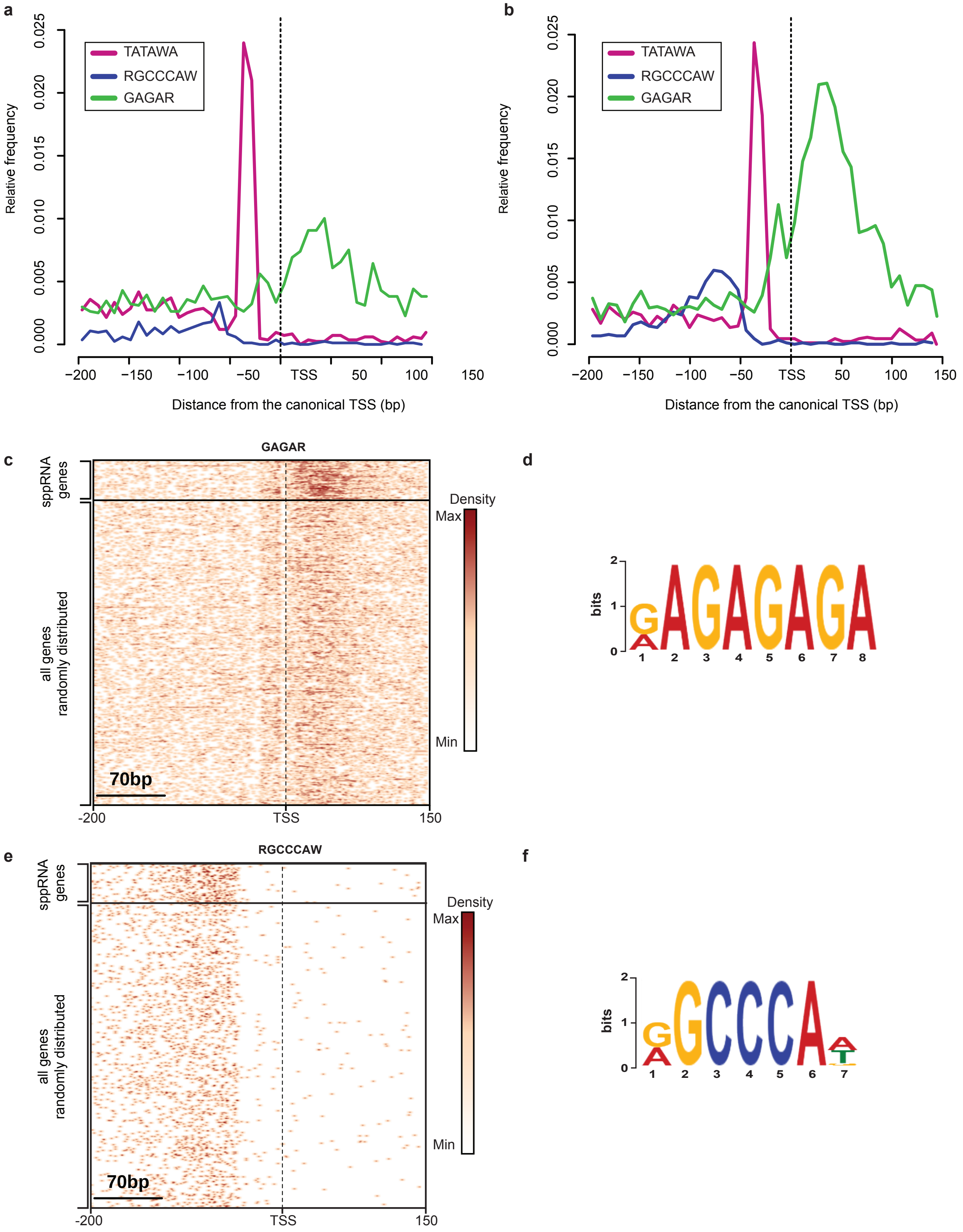

Supplementary Figure 13. GAGA-box and TCP binding motifs are enriched for sppRNA genes. **a** Relative frequency of three cis-element motifs TATA-box (TATAWA), TCP-binding motif (RCCCAW), GAGA-box (GAGAR) in a control set of genomic region 200bp upstream of the TSS and 150bp downstream of the TSS of a control set of genes with equal native expression distribution to sppRNA genes. **b** Relative frequency of three cis-elements motif TATA-box (TATAWA), TCP-binding motif (RGCCCAW), GAGA-box (GAGAR) in genomic region 200bp upstream of the TSS and 150bp downstream of the TSS of sppRNA genes (n=1173). **c** Heatmap of cis-element GAGA-box (GAGAR) in genomic regions 200 bp upstream and 150 bp downstream of the TSS of sppRNA genes (n=1155) and all non-overlapping genes from 75% top signal of nascent transcription (n=12116). **d** MEME suite Graphical representation of GAGA-box motif enriched in sppRNAs downstream regions. **e** Heatmap of TCP-binding motif (RGCCAW) in genomic regions 200 bp upstream and 150 bp downstream of the TSS of sppRNA genes (n=1155) and all non-overlapping genes from 75% top signal of nascent transcription (n=12116). The color scale in **(c-e)** represents motif frequency from low (white) to high (red). **f** MEME suite Graphical representation of TCP-binding motif enriched in sppRNA promoters.

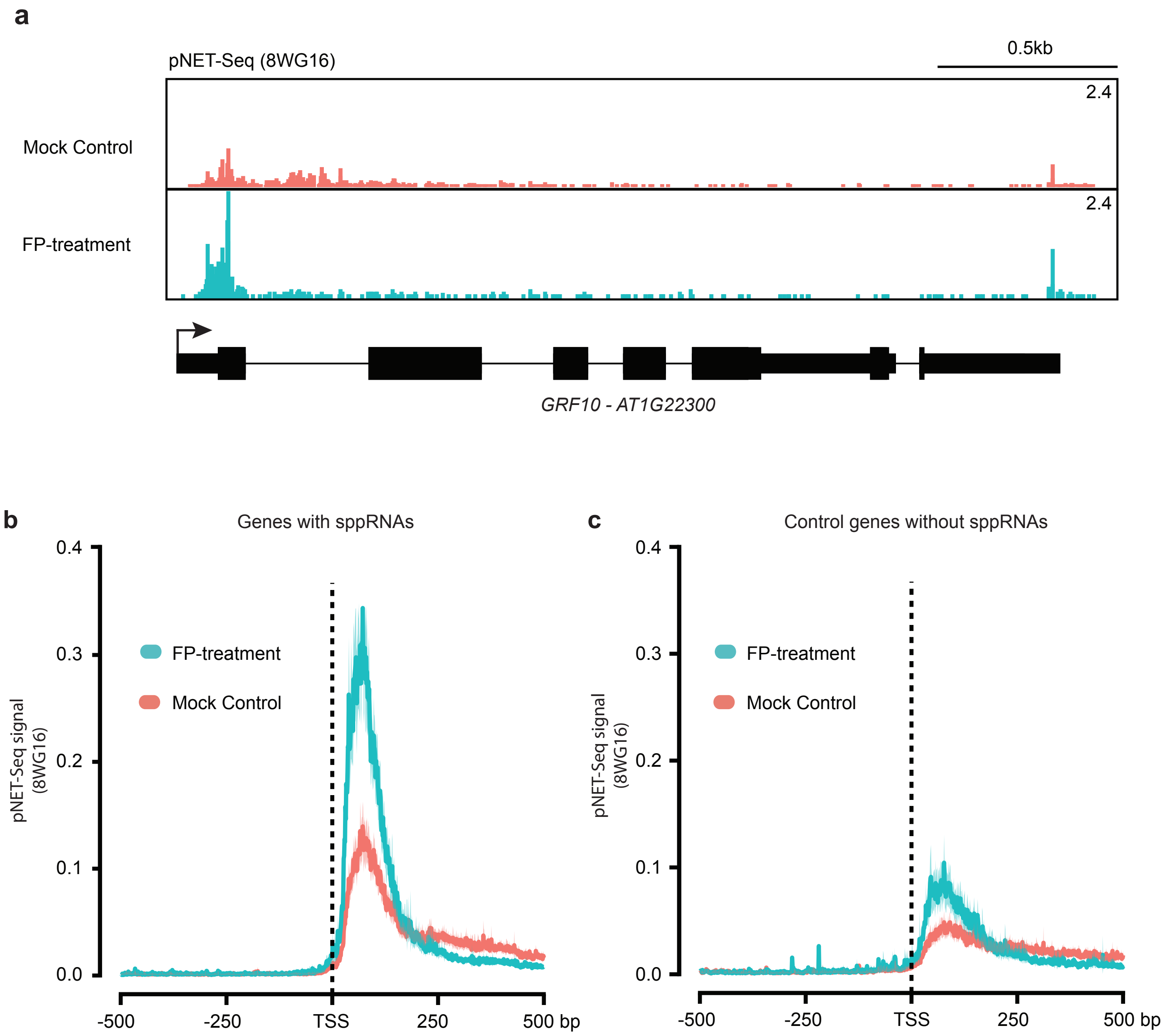

Supplementary Figure 14. Promoter-proximal RNAPII peaks at sppRNA-producing genes are highly sensitive to pTEF-b inhibitor Flavopiridol (FP). **a** Genome browser screenshot of pNET-seq signal along the sppRNA-containing gene *GRF10* (*AT1G22300*) following mock or FP treatment. **b-c** Metagene profiles of pNET-seq data from Arabidopsis seedlings before (mock) and after FP treatment (8) **(b)** Genes with sppRNAs; **(c)** Control set of genes without detectable sppRNAs but displaying equal nascent gene body transcription as those in panel **(b)**. Gene body is defined as +200 nts from annotated TSS and -200 nts from annotated PAS (see Methods). Shading represents the calculated confidence interval on the mean of 95%.

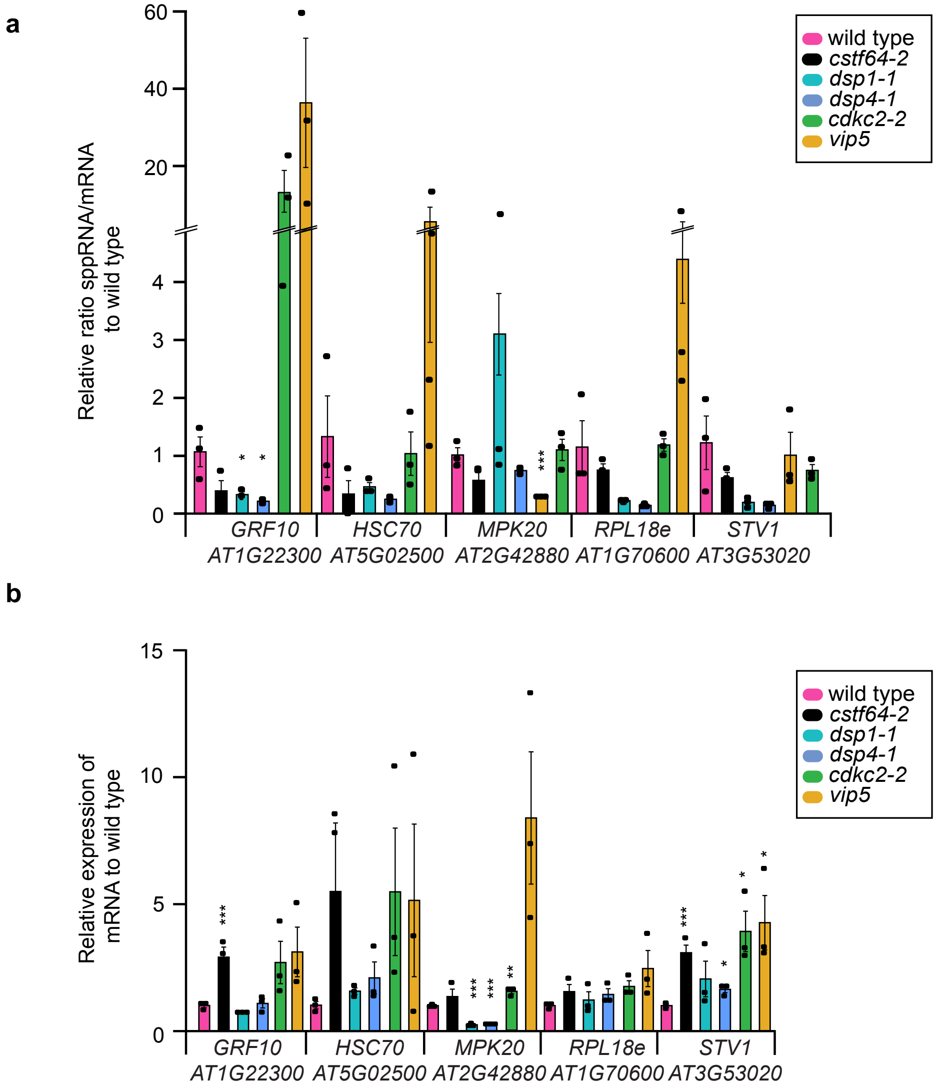

Supplementary Figure 15. RT-qPCR of sppRNA gene isforms in *Arabidopsis* elongation factor and 3´-end formation factor mutants. **a** sppRNA/mRNA ratio normalized to wild type for five sppRNA genes in wild type, *cdkc2-2*, *vip5*, *dsp1-1*, *dsp4-1* and *cstf64-2* by RT-qPCR. Black squares indicate individual data-points. Single asterisk denotes p<0.05, two asterisks denote p<0.01, while three asterisks denote p<0.001 between mutant and wild type by two sided Student's t-test. **b** RT-qPCR of mRNA expression normalized to wild type for five sppRNA genes in wild type, *cdkc2-2*, *vip5*, *dsp1-1*, *dsp4-1* and *cstf64-2* by RT-qPCR. Black squares indicate individual data-points. Single asterisk denotes p<0.05, two asterisks denote p<0.01, while three asterisks denote p<0.001 between mutants and wild type by a two sided Student's t-test. Data in **(a-b)** are presented as mean +/- SEM from three independent experiments. Source data of **(a-b)** are provided in the Source Data file.

Supplementary Table 1. TIF-Seq data sets. Important general values for all TIF-Seq datasets.

	WT(COL-0)	HEN2-2	WT(COL-0) 4°C	HEN2-2 4C	SSRP1	SPT16
nb of mapped read pairs	3270127	2796345	2379506	1625513	2274684	3172256
nb of clusters before mis-priming filtering	79842	73936	65055	46222	58777	78682
nb of detected genes	13505	13378	12671	10644	13735	13735
nb of clusters before TSS-Seq filtering	67537	62960	55348	39307	50439	67151
nb of detected genes	12697	12557	11946	9868	11890	12985
nb of clusters	45707	48762	42123	31998	40940	49901
nb of detected genes	10818	11328	10261	8920	10636	11528
nb of non overlapping genes used for the analysis	9048	9485	9595	7482	8901	9659
nb of unique genes with sppRNA	196	1274	71	1163	69	132
%sppRNA	2,17%	13,43%	0,74%	15,54%	0,78%	1,37%

Supplementary Table 2. Genotype lines described in the study.

Database Number	Genoytype	Source	Roles
N/A	Col-0	N/A	wild type
SMA1121	*hen2-2*	GABI_774H07 (9)	RNA helicase, part of TRAMP complex, degradation of ncRNAs
SMA0865	*spt16-1*	SAIL_392_G06 (10)	FACT subunit, RNAPII elongation, intragenic TSS suppression
SMA0870	*ssrp1-2*	SALK_001283c (10)	FACT subunit, RNAPII elongation, intragenic TSS suppression
SMA1448	*cdkc2-2*	SALK_029546; (11)	pTEF-b associated kinase, stimulates RNAPII elongation
SMA2449	*vip5*	SALK_062223 (12)	PAF1c subunit, stimulates RNAPII elongation
SMA2724	*cstf64-2*	SAIL_794_G11(13)	Required for 3'-end cleavage and polyadenylation of pre-mRNAs
SMA3496	*dsp1-1*	SALK_036691(14)	part of the plant integrator complex required for snRNA termination
SMA3497	*dsp1-4*	SALK_005904 (14)	part of the plant integrator complex required for snRNA termination

Supplementary References:

1. Kindgren, P., Ard, R., Ivanov, M. & Marquardt, S. Transcriptional read-through of the long non-coding RNA SVALKA governs plant cold acclimation. Nat Commun 9, 4561, (2018).
2. Nielsen, M. et al. Transcription-driven chromatin repression of Intragenic transcription start sites. PLoS Genet 15, e1007969, (2019).
3. Schurch, N. J. et al. Improved annotation of 3' untranslated regions and complex loci by combination of strand-specific direct RNA sequencing, RNA-Seq and ESTs. PLoS One, 9, e94270, (2014).
4. Pelechano, V., Wei, W., Jakob, P. & Steinmetz, L. M. Genome-wide identification of transcript start and end sites by transcript isoform sequencing. Nature protocols 9, 1740-1759, (2014).
5. Chew, O., Whelan, J. & Millar, A. H. Molecular definition of the ascorbate-glutathione cycle in Arabidopsis mitochondria reveals dual targeting of antioxidant defenses in plants. J Biol Chem , 278, 46869-46877, (2003).
6. Hornyik, C., Terzi, L. C. & Simpson, G. G. The spen family protein FPA controls alternative cleavage and polyadenylation of RNA. Dev Cell, 18, 203-213, (2010).
7. Zhao, X. et al. Global identification of Arabidopsis lncRNAs reveals the regulation of MAF4 by natural antisense RNA. Nat Commun, 9, 5056, (2018).
8. Zhu, J., Liu, M., Liu, X. & Dong, Z. RNA polymerase II activity revealed by GRO-seq and pNET-seq in Arabidopsis. Nat Plants 4, 1112-1123, (2018).
9. Lange, H. et al. The RNA helicases AtMTR4 and HEN2 target specific subsets of nuclear transcripts for degradation by the nuclear exosome in Arabidopsis thaliana. PLoS Genet **10**, e1004564, (2014).
10. Lolas, I. B. et al. The transcript elongation factor FACT affects Arabidopsis vegetative and reproductive development and genetically interacts with HUB1/2. Plant Journal **61**, 686-697, (2010).
11. Wang, Z. W., Wu, Z., Raitskin, O., Sun, Q. & Dean, C. Antisense-mediated FLC transcriptional repression requires the P-TEFb transcription elongation factor. Proceedings of the National Academy of Sciences of the United States of America **111**, 7468-7473, (2014)
12. Lu,C., Tian,Y., Wang,S., Su,Y.,Mao,T.,Huang,T.,Chen,Q.Xu,Z.,Ding,Y. Phosphorylation of SPT5 by CDKD;2 Is Required for VIP5 Recruitment and Normal Flowering in Arabidopsis thaliana. The Plant Cell 29(2), 277-291, (2017).
13. Liu, F., Marquardt, S., Lister, C., Swiezewski, S. & Dean, C. Targeted 3' processing of antisense transcripts triggers Arabidopsis FLC chromatin silencing. Science **327**, 94-97, (2010).
14. Liu, Y. et al. snRNA 3' End Processing by a CPSF73-Containing Complex Essential for Development in Arabidopsis. PLoS biology **14**, e1002571, (2016).
