# Supplementary material for: Transcript isoform sequencing reveals widespread promoter-proximal transcriptional termination in Arabidopsis
Source: Nat Commun. 2020 May 22;11:2589. doi: 10.1038/s41467-020-16390-7 (PMC7244574; doi:10.1038/s41467-020-16390-7)
Supplement: Supplementary file 3 — Description of Additional Supplementary Information [file 41467_2020_16390_MOESM3_ESM.pdf]

Description of Additional Supplementary Files

File Name: Supplementary Data 1
Description: Genes with sppRNA detected at 22C and GO-term enrichment results.

File Name: Supplementary Data 2
Description: Oligonucleotides used in the study.

File Name: Supplementary Data 3
Description: Genome coordinates of all sppRNAs clusters per TIF-Seq dataset.
