# Supplementary material for: Transcript isoform sequencing reveals widespread promoter-proximal transcriptional termination in Arabidopsis
Source: Nat Commun. 2020 May 22;11:2589. doi: 10.1038/s41467-020-16390-7 (PMC7244574; doi:10.1038/s41467-020-16390-7)
Supplement: Supplementary file 7 — Reporting summary [file 41467_2020_16390_MOESM7_ESM.pdf]

nature research

Corresponding author(s): Sebastian Marquardt

Last updated by author(s): Apr 6, 2020

Reporting Summary

Nature Research wishes to improve the reproducibility of the work that we publish. This form provides structure for consistency and transparency in reporting. For further information on Nature Research policies, seeAuthors & Referees and theEditorial Policy Checklist .

Statistics

For all statistical analyses, confirm that the following items are present in the figure legend, table legend, main text, or Methods section.

n/a	Confirmed	
☐	☒	The exact sample size (*n*) for each experimental group/condition, given as a discrete number and unit of measurement
☐	☒	A statement on whether measurements were taken from distinct samples or whether the same sample was measured repeatedly
☐	☒	The statistical test(s) used AND whether they are one- or two-sided<br>*Only common tests should be described solely by name; describe more complex techniques in the Methods section.*
☐	☒	A description of all covariates tested
☒	☐	A description of any assumptions or corrections, such as tests of normality and adjustment for multiple comparisons
☐	☒	A full description of the statistical parameters including central tendency (e.g. means) or other basic estimates (e.g. regression coefficient) AND variation (e.g. standard deviation) or associated estimates of uncertainty (e.g. confidence intervals)
☐	☒	For null hypothesis testing, the test statistic (e.g. *F*, *t*, *r*) with confidence intervals, effect sizes, degrees of freedom and *P* value noted<br>*Give P values as exact values whenever suitable.*
☒	☐	For Bayesian analysis, information on the choice of priors and Markov chain Monte Carlo settings
☒	☐	For hierarchical and complex designs, identification of the appropriate level for tests and full reporting of outcomes
☐	☒	Estimates of effect sizes (e.g. Cohen's *d*, Pearson's *r*), indicating how they were calculated

Our web collection on statistics for biologists contains articles on many of the points above.

Software and code

Policy information about availability of computer code

Data collection	all code used in the data analysis are available at https://github.com/qthom/planTIF .
Data analysis	all code used in the data analysis are available at https://github.com/qthom/planTIF . We used the following releases for analysis: IGV v2.3.93,Python v3.6.10,AfterQC v0.9.7,cutadapt v1.18,SAMtools 1.7,UMI-Tools v0.5.5,STAR v2.6.1c,R v3.4.1 and version associated packages (specifically, CAGEfightR v1.1.0, JASPAR2016 v1.12.0, seqPattern v1.16.0, TxDb.Athaliana.BioMart.plantsmart28 v3.2.2)

For manuscripts utilizing custom algorithms or software that are central to the research but not yet described in published literature, software must be made available to editors/reviewers. We strongly encourage code deposition in a community repository (e.g. GitHub). See the Nature Research guidelines for submitting code & software for further information.

Data

Policy information about availability of data

All manuscripts must include a data availability statement. This statement should provide the following information, where applicable:

- Accession codes, unique identifiers, or web links for publicly available datasets
- A list of figures that have associated raw data
- A description of any restrictions on data availability

Data are deposited to NCBI GEO under accession number GSE129523. The source data underlying the manuscript Figures are provided as Source Data file. Fig. 1a,2c-d,3g,6c and Supplementary figures 3e-f,5b-f,8f,9d use raw alignment files obtained from the analytic pipeline.

Field-specific reporting

Please select the one below that is the best fit for your research. If you are not sure, read the appropriate sections before making your selection.

[x] Life sciences   [ ] Behavioural & social sciences   [ ] Ecological, evolutionary & environmental sciences

For a reference copy of the document with all sections, see nature.com/documents/nr-reporting-summary-flat.pdf

Life sciences study design

All studies must disclose on these points even when the disclosure is negative.

Sample size	Each genotype TIF-Seq was performed in duplicates. We estimated the needed RNA starting amount and NGS reads for the Arabidopsis TIF-seq data based on previous studies in budding yeast.
Data exclusions	No data was excluded
Replication	We used the biological repeat TIF-seq data to verify experimental findings. Our TIF-seq analysis pipeline considers new transcripts isoforms as real when they are detectable in both repeats. Some isoforms are only detectable in one of the libraries, particularly for genes with low expression level. These transcrips may also represent real transcript isoforms yet we have excluded them from the analysis. Additionally, the transcripts in TIF-Seq data were filtered when there were no exact match with peaks detected in TSS-Seq in the same genotype.
Randomization	We allocated mutants or environmental treatments in groups and compared the TIF-seq data to a wild type reference data set. When comparing WT to mutants, A random group of equal distribution in pNET-Seq (i.e fig. 5a,c) or corresponding data set. (fig. 6c-d) was determined for each to allow comparison.
Blinding	Our experimentation design targeted a comparison of wild type TIF-seq data to mutant or environmental treatment data. Blinding does not apply to our study design.

Reporting for specific materials, systems and methods

We require information from authors about some types of materials, experimental systems and methods used in many studies. Here, indicate whether each material, system or method listed is relevant to your study. If you are not sure if a list item applies to your research, read the appropriate section before selecting a response.

Materials & experimental systems

n/a	Involved in the study	
[x]	[ ]	Antibodies
[x]	[ ]	Eukaryotic cell lines
[x]	[ ]	Palaeontology
[x]	[ ]	Animals and other organisms
[x]	[ ]	Human research participants
[x]	[ ]	Clinical data

Methods

n/a	Involved in the study	
[x]	[ ]	ChIP-seq
[x]	[ ]	Flow cytometry
[x]	[ ]	MRI-based neuroimaging
